
JOURNAL INFORMATION
==============================
NLM Title Abbreviation: ScientificWorldJournal
Journal ID: ScientificWorldJournal
NLM Journal ID: 101131163
Journal ID: TSWJ

The Scientific World Journal

ISSN: 2356-6140
EISSN: 1537-744X
Publisher: Wiley

ARTICLE INFORMATION
==============================
PMCID: PMC5763889
PMID: 20602081
DOI: 10.1100/tsw.2010.101
Article ID: 914190
Article version: 1
Subjects: Directions in Science

New Surprises from the Deep — The Family of Small Regulatory RNAs Increases

Wittmann Jürgen *
Jäck Hans-Martin
Division of Molecular Immunology, Department of Internal Medicine III, Nikolaus-Fiebiger-Center of Molecular Medicine, Friedrich-Alexander-University Erlangen-Nürnberg, Erlangen, Germany
*Jürgen Wittmann: jwittman@molmed.uni-erlangen.deAcademic Editor: Adele Murrell

Publication date: 2010
Electronic publication date: 2010 Jun 29
Volume: 10
First page: 1239
Last page: 1243
Received 2010 Feb 25; Revised 2010 Apr 13; Accepted 2010 Apr 30
Copyright: Copyright © 2010 Jürgen Wittmann and Hans-Martin Jäck.
Copyright year: 2010
License: This is an open access article distributed under the Creative Commons Attribution License, which permits unrestricted use, distribution, and reproduction in any medium, provided the original work is properly cited.
License URL: https://creativecommons.org/licenses/by/3.0/

Keywords: deep sequencing, miRNA, ncRNA, snoRNA, tRNA

Funding: Deutsche Forschungsgemeinschaft JA 968/4 University Hospital of the University of Erlangen-Nürnberg Hiege Foundation Hamburg University of Erlangen-Nürnberg

==============================
Directions in Science
*TheScientificWorldJOURNAL* (2010) 10, 1239–1243
ISSN 1537-744X; DOI 10.1100/tsw.2010.101

TheScientificWorldJOURNAL
www.thescientificworld.com

### New Surprises from the Deep — The Family of Small Regulatory RNAs Increases

Jürgen Wittmann* and Hans-Martin Jäck

*Division of Molecular Immunology, Department of Internal Medicine III, Nikolaus-Fiebiger-Center of Molecular Medicine, Friedrich-Alexander-University Erlangen-Nürnberg, Erlangen, Germany*

**E-mail**: jwittman@molmed.uni-erlangen.de, hjaeck@molmed.uni-erlangen.de

*Received February 25, 2010; Revised April 13, 2010; Accepted April 30, 2010; Published June 29, 2010*

**KEYWORDS**: deep sequencing, miRNA, ncRNA, snoRNA, tRNA

The amount of knowledge about new classes of small RNA molecules has expanded at a breathtaking pace in the last few years. This expansion is mainly due to the advent of new, advanced, Next-Generation Sequencing (NGS) technologies using massive parallel sequencing on platforms such as the Illumina Genome Analyzer, ABI SOLiD, or Roche/454 Genome Sequencer FLX[1]. The likelihood of a molecule being sequenced correlates with its abundance and, until recently, small rare RNA species besides very abundant small RNAs had only a little chance of being discovered because of the small-scale sequencing approaches used. The vast amounts of data presently generated and coupled with computational analysis allow a yet unprecedented exploration of the heterogeneity of 20- to 30-nt-long small RNAs and their role in post-transcriptional gene regulation. The continual discovery of new small RNA species suggests that we are only just beginning to appreciate the complexity of small RNAs and their functions. An overview of the molecules discussed in this article is summarized in Table 1 and Fig. 1.

The first and largest group of small RNA molecules analyzed thus far consists of microRNAs (miRNAs). First described in 1993 by Lee and colleagues[5], miRNAs have been the focus of intensive investigations in recent years. miRNAs are single-stranded RNA molecules about 22 nucleotides (nt) in length. Their biogenesis begins with the transcription of miRNA genes to generate primary (pri-) miRNA transcripts in the nucleus. These pri-miRNAs are noncoding RNA, i.e., they are not translated into protein. Parts of the pri-miRNA transcripts have short, imperfectly base-paired, stem-loop structures containing what will become the mature miRNA. These hairpin structures are released from the pri-miRNAs by Drosha, a nuclear RNaseIII enzyme, together with its "ruler", DGCR8, as ~70-nt fragments called precursor (pre-) miRNAs. Pre-miRNAs are transported into the cytoplasm by Exportin 5, where they are further processed by Dicer, a second RNaseIII enzyme that cuts off the terminal loop and generates an imperfect, double-stranded RNA duplex[6]. This duplex binds to proteins of the Argonaute family as part of the RNA-induced silencing complex (RISC). After unwinding the duplex and discarding the passenger strand, the RISC complex containing the remaining leading strand binds to the target mRNA based on sequence complementarity, either by perfect base-pairing leading to direct mRNA cleavage or, predominantly, by imperfect base-pairing leading mostly to translational repression[7]. Each miRNA regulates the expression of up to several hundred mRNAs and, conversely, one mRNA can be regulated by multiple miRNAs. miRNAs can therefore fine tune the expression of a large battery of genes, leading to subtle changes resulting in a large effect. In contrast, miRNAs can also cause a significant decrease in the abundance of a

*Corresponding author.
©2010 with author.
Published by TheScientificWorld; www.thescientificworld.com

**TABLE 1**
**Summary of Small Endogenous Regulatory RNAs and Their Potential Functions**

| RNA Type | Functions | Ref. |
|---|---|---|
| miRNA | • Processed by endonucleases Drosha and Dicer from up to several thousand nt-long pri-miRNAs and ~70- to 90-nt-long pre- miRNAs, respectively<br>• Post-transcriptional regulators by guiding RISC to mRNA, which either interferes with translation or induces mRNA degradation<br>• Expressed in all tissues and cell types of metazoa | [6,7] |
| snoRNA | • Processed by Dicer from longer precursors<br>• Controls modifications such as the 2'-O-methylation and pseudouridylation of rRNAs, snRNAs, and possibly other RNAs<br>• Localized in nucleoli and Cajal bodies in eukaryotic cells | [2] |
| tRFs | • Processed from different mature tRNAs by specific endonucleases (e.g., ELAC2 and Dicer) under stress response and possibly others occasions<br>• Competition with miRNA for components of the RISC? | [4] |
| piRNA | • Silencing of different classes of retroelements in germ line cells<br>• Transcription and processing of precursor unknown | [12] |
| snRNA | • Localized in nuclei of eukaryotic cells<br>• Incorporated in snRNP complexes<br>• Control splicing of pre-mRNAs<br>• Regulates transcription by controlling the elongation factor P-TEFb<br>• Expressed in all tissues and cell types of eukaryotes | [3] |

*Note:* snoRNA: small nucleolar RNA; tRFs: tRNA-derived RNA fragments; piRNA: Piwi-interacting RNA; snRNA: small nuclear RNA; snRNP: small nuclear ribonucleoprotein.

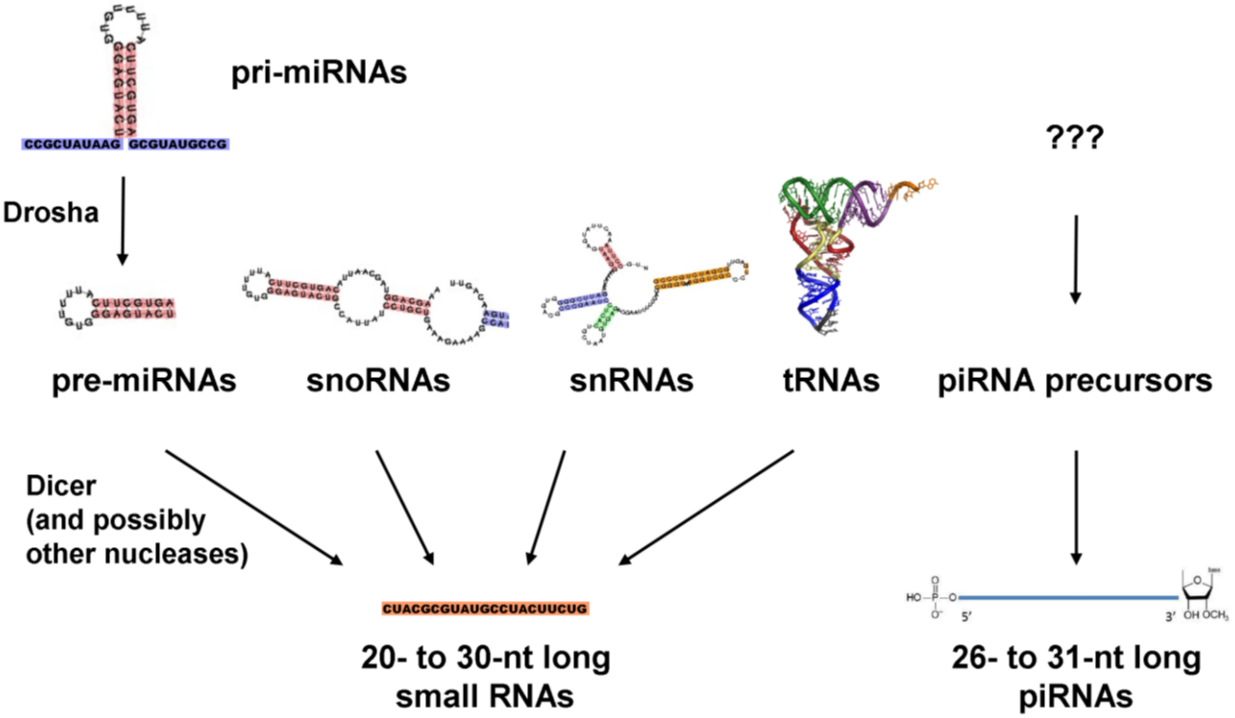

**FIGURE 1.** A variety of RNA classes is processed into 20- to 30-nt-long small RNAs. For details see text and Table 1. Small RNAs are not drawn to scale. snRNA and snoRNA pictures are taken from the Rfam database, while the tRNA structure was reproduced with the kind permission of N.R. Voss.

single transcript, affecting the expression of an individual gene expression as much as a transcriptional repressor. Therefore, it is not surprising that miRNAs have been shown to be involved in all types of cancer investigated thus far, either as modulators of the expression of cancer-related genes or as tumor-suppressor genes or oncogenes themselves[8].

Genome-wide profiling of miRNAs by high-throughput sequencing usually starts with the fractionation of total cellular RNA into a pool of 20- to 30-nt-long small RNAs. Small RNAs other than *bona fide* miRNAs are usually detected by high-throughput sequencing in the 20- to 30-nt-long RNA pool. Until recently, these fragments have been neglected as degradation products without any biological function. Now, several recent studies, such as those of Kawaji et al.[9], Babiarz et al.[10], or Cole et al.[11], change this picture and open a new avenue on the function of small RNAs that are generated from larger precursors. Some of the small RNA fragments could, for instance, derive from transcription of overlapping genes, potentially by usage of bidirectional promoters or by cleavage of folded RNAs harboring dsRNA segments. One example of small RNA fragments that derive from a known class of larger RNAs with a defined biosynthetic pathway is the class of small nuclear RNAs (snRNAs). snRNAs are about 100–300 nt in length, are incorporated in small nuclear ribonucleoprotein (snRNP) complexes, and function in the splicing of pre-mRNAs. The presence of short snRNA fragments in total cellular RNA preparations enriched for 20- to 30-nt-long RNA fragments suggests either a controlled cleavage of snRNA or the stabilization of the smaller snRNA fragments.

Piwi-interacting RNAs (piRNAs) are another class of small RNAs that are mainly expressed in ovaries and testes of different organisms. Their function in retrotransposon silencing is currently being elucidated and covered by several excellent reviews (e.g., Aravin et al.[12]).

Small nucleolar RNAs (snoRNAs) are usually between 60 and 300 nt long and mainly contribute to RNA modifications such as the 2’-O-methylation and pseudouridylation of ribosomal RNAs (rRNAs), snRNAs, and, putatively, other RNAs. snoRNAs are localized in nucleoli and Cajal bodies in eukaryotic cells. Expression profiling of fractions containing 20- to 30-nt-long RNAs identified distinct cleavage products of snoRNAs[13], and recent reports have demonstrated that components of the RNA silencing pathway associate with these structures and that snoRNAs can be processed into miRNA-like RNAs in several organisms[14]. Ender et al.[15] reported that snoRNA fragments are associated with human Ago1 and Ago2 proteins, and that processing of the snoRNA precursor requires Dicer activity. Importantly, they could show that these small fragments have functional mRNA targets, suggesting a role for the snoRNA-processing products in post-transcriptional gene silencing similar to that of miRNAs. Similar findings were reported for the primitive eukaryote *Giardia lamblia*, indicating conservation of this snoRNA-derived miRNA-mediated function[16]. An evolutionary relationship between miRNAs and snoRNAs was demonstrated recently, as several miRNA precursors show significant similarity to and features typical of snoRNAs[17]. Interestingly, a snoRNA encoded in the genome of Epstein-Barr virus (EBV) has also recently been reported[18]. The EBV snoRNA is processed into 24-nt-sized RNA species and serves as an miRNA-like precursor, possibly regulating viral mRNAs. In summary, the evolutionary conservation and the similarity in the functions of snoRNA-derived small RNAs and miRNAs warrants a more detailed investigation of the role of snoRNAs in carcinogenesis and development.

The detection of transfer RNA (tRNA) fragments in deep-sequencing analyses of RNA preparations enriched for 20- to 30-nt-long fragments was unexpected and prompted further investigations[9]. Exciting recent studies addressed this point and identified, under stress and also under normal conditions, different tRNA processing pathways that generate tRNA-derived RNA fragments, so-called tRFs. This indicates that a tRNA could have new functions besides its role in decoding mRNA into polypeptides[11,19,20]. It has been known for a long time that tRNA cleavage occurs in bacteria as a response to bacteriophage infection[21]. Recent work has demonstrated that a conserved response to stress includes the endonucleolytic cleavage of cytoplasmic tRNAs by ribonucleases that are normally secreted or sequestered[22,23]. This stress-induced tRNA cleavage depends on angiogenin, a member of the RNase A family[23,24]. Several lines of evidence imply that the observed cleavage products are not simply degradation products. First, the cleavage is not limited to specific tRNAs and occurs primarily in the anticodon loop. Second, the amount of full-length tRNAs does not decline significantly and the abundance of tRNA fragments is much lower than that of full-length tRNAs, which makes a defense mechanism to deplete the cellular tRNA pool unlikely. In addition, only fully mature tRNAs seem to be a substrate for the processing, as no intron-containing tRNA fragments have been reported. Finally, the 5' ends of the tRNA fragments often include the 3' terminal CCA sequence of a mature tRNA.

Recent studies by Cole and colleagues[11] now show that the generation of small tRNA fragments can also depend on the classical RNA interference pathway, i.e., the *in vivo* production of tRF depends on Dicer and Dicer cleaves tRNAs *in vitro*. These results are consistent with findings of Lee et al.[19], who also reported that tRFs are precise processing products from the 5' or 3' end of mature or precursor tRNAs and constitute a class of short RNAs that are second most abundant to miRNAs. One of the more closely examined tRFs was well expressed in several cancer cell lines, but much less in normal tissues, and its expression in cell lines was tightly correlated with cell proliferation. Small interfering RNA (siRNA)–mediated knockdown of the tRF resulted in impaired cell proliferation with accumulation of cells in the G2 phase of the cell cycle. The finding that tRFs are generated in the cytoplasm by the tRNA 3' endonuclease ELAC2 (elaC homolog 2), a known prostate cancer susceptibility gene, further supports a role of tRFs in tumorigenesis. tRFs and their processing by ELAC2 or Dicer were also reported by Haussecker and colleagues[20]. The authors showed that tRFs, regardless of their processing pathway, localize to the cytoplasm and, in contrast to miRNAs, preferentially associate with Argonaute 3 and 4. The authors speculated that competition between different classes of small RNAs, e.g., tRFs and miRNAs, for components of the RISC might reciprocally regulate their global activity.

tRNA endonucleases also play a role in the processing of miRNA precursors bearing a 5' tRNA moiety. Such chimeric tRNA-miRNAs exist in the genome of the mouse-γ herpesvirus 68. Transcribed viral tRNA-like sequences are not cleaved by the canonical miRNA processor Drosha, but rather liberated by the endonuclease tRNase Z into a pre-miRNA hairpin, which can then enter the canonical miRNA processing pathway[25]. Collectively, these findings suggest that small tRNA fragments are not random by-products of tRNA degradation or biogenesis, but are an abundant and novel class of short RNAs with a precise sequence structure. They can be generated by different means and show specific expression patterns, possibly implying specific biological roles in as yet unappreciated cellular functions.

Extended studies on these new exciting classes of small RNAs, which were previously thought to be mere degradation products, will undoubtedly uncover novel roles for these tiny molecules in health and disease.

### ACKNOWLEDGMENTS

This work was supported, in part, by the Deutsche Forschungsgemeinschaft (DFG) through research grant FOR832 (JA 968/4) to H.-M.J., the Interdisciplinary Center for Clinical Research (IZKF, project D7) at the University Hospital of the University of Erlangen-Nürnberg and the Hiege Foundation Hamburg to H.-M.J. and J.W., as well as the intramural ELAN Fonds at the University Hospital of the University of Erlangen-Nürnberg to J.W.

### REFERENCES

1. Mardis, E.R. (2008) Next-generation DNA sequencing methods. *Annu. Rev. Genomics Hum. Genet.* **9**, 387–402.
2. Dieci, G., Preti, M., and Montanini, B. (2009) Eukaryotic snoRNAs: a paradigm for gene expression flexibility. *Genomics* **94**, 83–88.
3. Patel, S.B. and Bellini, M. (2008) The assembly of a spliceosomal small nuclear ribonucleoprotein particle. *Nucleic Acids Res*. **36**, 6482–6493.
4. Thompson, D.M. and Parker, R. (2009) Stressing out over tRNA cleavage. *Cell* **138**, 215–219.
5. Lee, R.C., Feinbaum, R.L., and Ambros, V. (1993) The C. elegans heterochronic gene lin-4 encodes small RNAs with antisense complementarity to lin-14. *Cell* **75**, 843–854.
6. Winter, J., Jung, S., Keller, S., Gregory, R.I., and Diederichs, S. (2009) Many roads to maturity: microRNA biogenesis pathways and their regulation. *Nat. Cell Biol.* **11**, 228–234.

7. Bartel, D.P. (2009) MicroRNAs: target recognition and regulatory functions. *Cell* **136**, 215–233.
8. Esquela-Kerscher, A. and Slack, F.J. (2006) Oncomirs - microRNAs with a role in cancer. *Nat. Rev. Cancer* **6**, 259–269.
9. Kawaji, H., Nakamura, M., Takahashi, Y., Sandelin, A., Katayama, S., Fukuda, S., Daub, C.O., Kai, C., Kawai, J., Yasuda, J., Carninci, P., and Hayashizaki, Y. (2008) Hidden layers of human small RNAs. *BMC Genomics* **9**, 157.
10. Babiarz, J.E., Ruby, J.G., Wang, Y., Bartel, D.P., and Blelloch, R. (2008) Mouse ES cells express endogenous shRNAs, siRNAs, and other Microprocessor-independent, Dicer-dependent small RNAs. *Genes Dev*. **22**, 2773–2785.
11. Cole, C., Sobala, A., Lu, C., Thatcher, S.R., Bowman, A., Brown, J.W., Green, P.J., Barton, G.J., and Hutvagner, G. (2009) Filtering of deep sequencing data reveals the existence of abundant Dicer-dependent small RNAs derived from tRNAs. *RNA* **15**, 2147–2160.
12. Aravin, A.A., Hannon, G.J., and Brennecke, J. (2007) The Piwi-piRNA pathway provides an adaptive defense in the transposon arms race. *Science* **318**, 761–764.
13. Ro, S., Song, R., Park, C., Zheng, H., Sanders, K.M., and Yan, W. (2007) Cloning and expression profiling of small RNAs expressed in the mouse ovary. *RNA* **13**, 2366–2380.
14. Taft, R.J., Glazov, E.A., Lassmann, T. Hayashizaki, Y., Carninci, P., and Mattick, J.S. (2009) Small RNAs derived from snoRNAs. *RNA* **15**, 1233–1240.
15. Ender, C., Krek, A., Friedländer, M.R., Beitzinger, M., Weinmann, L., Chen, W., Pfeffer, S., Rajewsky, N., and Meister, G. (2008) A human snoRNA with microRNA-like functions. *Mol. Cell* **32**, 519–528.
16. Saraiya, A.A. and Wang, C.C. (2009) snoRNA, a novel precursor of microRNA in Giardia lamblia. *PLoS Pathog*. **4**, e1000224.
17. Scott, M.S., Avolio, F., Ono, M., Lamond, A.I., and Barton, G.J. (2009) Human miRNA precursors with box H/ACA snoRNA features. *PLoS Comput. Biol*. **5**, e1000507.
18. Hutzinger, R., Feederle, R., Mrazek, J., Schiefermeier, N., Balwierz, P.J., Zavolan, M., Polacek, N., Delecluse, H.J., and Huttenhofer, A. (2009) Expression and processing of a small nucleolar RNA from the Epstein-Barr virus genome. *PLoS Pathog.* **5**, e1000547.
19. Lee, Y.S., Shibata, Y., Malhotra, A., and Dutta, A. (2009) A novel class of small RNAs: tRNA-derived RNA fragments (tRFs). *Genes Dev*. **23**, 2639–2649.
20. Haussecker, D., Huang, Y., Lau, A., Parameswaran, P., Fire, A.Z., and Kay, M.A. (2010) Human tRNA-derived small RNAs in the global regulation of RNA silencing. *RNA* **16**, 673–695.
21. Levitz, R., Chapman, D., Amitsur, M., Green, R., Snyder, L., and Kaufmann, G. (1990) The optional E. coli prr locus encodes a latent form of phage T4-induced anticodon nuclease. *EMBO J.* **9**, 1383–1389.
22. Thompson, D.M., Lu, C., Green, P.J., and Parker, R. (2008) tRNA cleavage is a conserved response to oxidative stress in eukaryotes. *RNA* **14**, 2095–2103.
23. Yamasaki, S., Ivanov, P., Hu, G.F., and Anderson, P. (2009) Angiogenin cleaves tRNA and promotes stress-induced translational repression. *J. Cell Biol*. **185**, 35–42.
24. Fu, H., Feng, J., Liu, Q., Sun, F., Tie, Y., Zhu, J., Xing, R., Sun, Z., and Zheng, X. (2009) Stress induces tRNA cleavage by angiogenin in mammalian cells. *FEBS Lett.* **583**, 437–442.
25. Bogerd, H.P., Karnowski, H.W., Cai, X., Shin, J., Pohlers, M., and Cullen, B.R. (2010) A mammalian herpesvirus uses noncanonical expression and processing mechanisms to generate viral microRNAs. *Mol. Cell* **37**, 135–142.

**This article should be cited as follows:**

Wittmann, J. and Jäck, H.-M. (2010) New surprises from the deep — the family of small regulatory RNAs increases. *TheScientificWorldJOURNAL* **10**, 1239–1243. DOI 10.1100/tsw.2010.101.
